# An Examination of Not-For-Profit Stakeholder Networks for Relationship Management: A Small-Scale Analysis on Social Media

**DOI:** 10.1371/journal.pone.0163914

**Published:** 2016-10-06

**Authors:** Jessica Wyllie, Benjamin Lucas, Jamie Carlson, Brent Kitchens, Ben Kozary, Mohamed Zaki

**Affiliations:** 1 Newcastle Business School, University of Newcastle, Callaghan, Australia; 2 Department of Marketing and Supply Chain Management, School of Business and Economics, Maastricht University, Maastricht, Netherlands and BISS Institute, Maastricht, Netherlands; 3 McIntire School of Commerce, University of Virginia, Charlottesville, Virginia, United States of America; 4 Forethought, Melbourne, Australia; 5 Cambridge Service Alliance, Insitute for Manufacturing, Department of Engineering, University of Cambridge, Cambridge, United Kingdom; Nankai University, CHINA

## Abstract

Using a small-scale descriptive network analysis approach, this study highlights the importance of stakeholder networks for identifying valuable stakeholders and the management of existing stakeholders in the context of mental health not-for-profit services. We extract network data from the social media brand pages of three health service organizations from the U.S., U.K., and Australia, to visually map networks of 579 social media brand pages (represented by nodes), connected by 5,600 edges. This network data is analyzed using a collection of popular graph analysis techniques to assess the differences in the way each of the service organizations manage stakeholder networks. We also compare node meta-information against basic topology measures to emphasize the importance of effectively managing relationships with stakeholders who have large external audiences. Implications and future research directions are also discussed.

## Introduction

In recent years, healthcare and not-for-profit organizations have turned to social media platforms (e.g. Facebook and Twitter) to engage, communicate and collaborate with their various stakeholders; for example, to undertake research, promote causes, and educate consumers of their health services and programs [1]. At the same time, both present and future consumers of health services are increasingly searching for health information online [2]. Thus, valuable network data is being generated in the online environment, creating an important resource for studying stakeholder networks.

Not-for-profit healthcare organizations have become increasingly dependent upon a diverse network of stakeholder groups (e.g. referring clinicians and providers, politicians, for-profit companies, celebrities and media personalities and patients), to help market and build awareness of their services [3]. By understanding these stakeholder networks, the various roles they play, and the influence they exert—whether through ownership or formal partnerships—healthcare organizations are able to advance service provision [4]. Further opportunities also arise to create inter-organizational mutual value by fine-tuning their marketing tactics, resource allocation [5], and strategies for innovation [6,7], and knowledge sharing [8].

Despite the importance of social media stakeholder networks, health organizations (and organizations generally) are not always aware of the exact composition and structure of their social media stakeholder networks, and to what extent stakeholders are passive or active within the network [9]. Consequently, healthcare and not-for-profit organizations are failing to maximize the utility of the interactive functions of social media and engage the range of key stakeholders within their networks, where opportunities for mutual value creation are identified and exploited [10,11]. As such, we propose that social media stakeholder networks should feature more prominently in social media analytics for marketers generally, but especially for health services, not-for-profit and cause-focused organizations. We hope to foster more research in the area of business landscape analysis in online environments [12] and understanding stakeholder engagement in networks within health service contexts [13].

More broadly, studying the development of service networks and how data can be used to advance service provision has been highlighted as a high-priority topic [14]. The academic discussion around service networks has also grown recently to include the notion of service ecosystems. Where over less immediate time horizons networks of stakeholders co-produce service contexts and end experiences which represents a landscape conducive to innovation [14, 15,16]. In service science, data-driven research has been recognized as a way for service managers to unlock opportunities in the new data-rich business environment, with a significant body of research building on the subject [17, 18, 19, 20].

Methodologically speaking, using network analysis techniques to study stakeholder networks and generate this understanding is not a new idea [21]. However, advances in complex network analysis in computer science and the availability of new sources of data, such as social media data, afford researchers new freedom in conducting such research. For example, data scientists have used network analysis to study a variety of human behaviors on social media at a large scale including; social contagion [22], rumor cascades [23], emotional contagion [24], tie strength [25], social media interactions and geographic location prediction [26], and relationship status [27].

In this study, we develop and illustrate a simple, data-driven approach as a pathway to understanding stakeholder networks via social media for healthcare and not-for-profit organizations. Such data-driven approaches unlock opportunities for value creation by mapping and analyzing social media stakeholders that are ‘valuable’ to monitor, engage and/or establish a relationship with. In doing so, the ultimate goal is to improve service provision for end users. This notion is generally consistent with the modern idea of customer experience management, under which market data is continually monitored, with insights fed back into service development [28].

Our proposed approach involves ‘ranking’ and visualizing constituent stakeholders based on their connections to other stakeholders using basic network topology measures (i.e. degree centrality and Eigenvector centrality), which are then extended by conducting a graph reduction exercise (implementing a minimum spanning tree) to expose important ‘stakeholder hubs’ within networks, using Facebook as our research site. Drawing from three Facebook brand pages from mental health organizations in the U.S., U.K., and Australia, we compare and contrast social media stakeholder networks between the three organizations. Specifically, we aim to answer two broad research questions:

1. To what extent can small-scale stakeholder network analysis on social media reveal useful relationship management insights for not-for-profit mental health service organizations?
2. How feasible are the implementation and ongoing use of tools (i.e. data extraction and graph generation) facilitating such analysis for not-for-profit mental health service organizations?

## Materials and Methods

### Data Extraction

We focus our study on not-for-profit mental health service organizations. Mental health disorders account for 14% of disease burden worldwide [29], and have therefore been recognized as a high-priority issue by governments around the world.

The dataset used in this study comprises publicly accessible Facebook brand page networks. Facebook now has 1.59 billion active users, and is widely used by organizations of all types to connect with consumers [30]. We focus on three organizations, which we use as seeds to extract networks: (1) Mental Health America (MHA, USA); (2) Mind (UK); and (3) Beyond Blue (Australia)—with all three sharing goals of raising awareness, promoting understanding, and improving the service of mental health. Of the three organizations, MHA and Mind have face-to-face affiliates (i.e. branches) in geographical county locations around their respective countries, whereas Beyond Blue operates a range of online services.

In summary, our dataset contains 579 social media brand pages (represented by nodes), connected by a total of 5600 edges, across three independent networks seeded from the brand pages of three not-for-profit mental health service organizations from three different international markets. We deliberately restricted the scale of our study, as smaller and focused studies in health can produce useful insights in very targeted and focused contexts [31].

We gathered our data using NetVizz, which is designed for collating social media API data into network files and ensuring parametrization [32]. The data were extracted via the Facebook API on April 3, 2015, whereby directed, unweighted graphs were produced. These graphs depict brand pages as nodes, and connections (i.e. page ‘likes’) between pages are represented by edges. Directionality is based upon the source of the page ‘like’ (i.e. which user initiated the connection). To reduce the complexity of the network, thereby enhancing the focus and maximizing the interpretability of the results of our analysis, we restricted our data capture to first-level connections (i.e. nodes other than the seed node are only included if they are connected to the seed node).

### Graph Analysis

We implement analysis and visualization using Gephi [33] consistent with similar recent research [34]. To understand and compare the structure of the three stakeholder networks, we divide analysis into five main steps which are discussed as follows.

#### (1) Network graph properties and structure

First, we assess the properties and structure of the network by looking at graph topology. Specifically, we employ degree centrality and Eigenvector centrality topology measures [35, 36] as a basis for fundamental comparison between networks. Degree centrality represents the number of inward and outward connections of any given node, whilst Eigenvector centrality, in basic terms, adds weighting to this calculation (See: [37] for another social network research application of this measure). Nodes are assigned scores deriving from both immediate and subsequent neighboring nodes within a network, with the calculation rewarding ‘hub’ nodes that connect to other ‘hub’ nodes [38].

#### (2) Community detection

Second, we use modularity to assess the extent to which our graphs can be partitioned, with m > 0.5 indicative of higher divisibility [39,40]. Gephi offers implementation of the algorithm described in Blondel et al. [41]. We use community detection to provide a general overview of network graph structure, as opposed to finding ‘hard’ community detection or cluster solutions. This procedure also helps enhance the visual presentation of the network graphs, by mathematically separating tightly-connected network sub-sections.

#### (3) Network graph reduction

Third, we reduce the network graphs using a minimum-spanning-tree (MST) procedure implemented using a Gephi plugin. The MST procedure produces a graph where all nodes are linked to each other via a shortest path solution [42, 43]. Consequently, we can analyze the fundamental structure of the network as well as ‘hubs’ within the network, which occur where brands perform a role of linking a family of nodes to the center of the graph. MST link reduction techniques help researchers to identify and isolate structural saliency in networks [44, 45] and have also been built upon for more formalized graph-partitioning (e.g. the MST-kNN algorithm, implemented in [46]).

#### (4) Pairwise network graph comparison

Next, we implement graphlet-heuristic-based pairwise network comparison using GraphCrunch 2 (outlined in [47]), to provide an assessment of network topological similarity/dissimilarity, as well as cross-validating our previous analysis steps [48, 49, 50, 51]. GraphCrunch 2 works with simple, unweighted, undirected networks. In this study, we report graphlet degree distribution (GDD) agreement, and relative graphlet frequency (RGF) distance, to assess local-level topological similarity. We also report Pearson and Spearman correlations of network degree distribution and path difference statistics to supplement this overview of network pairings.

#### (5) Network graph meta-information assessment

Finally, we incorporate node (i.e. brand) meta-information into the analysis, which is presented as a separate section within the study. This step involves comparing network graph topology measures (see Step 1), with the social media data linked to each brand in our dataset. In doing so, we can assess the relative ‘importance’ of nodes within their focal networks, which also enables comparison with the ‘importance’ of the nodes in their external networks. Such a comparison is, in our belief, a simple but highly effective tool for marketers to evaluate present and future stakeholder relationships.

**Fig 1:**
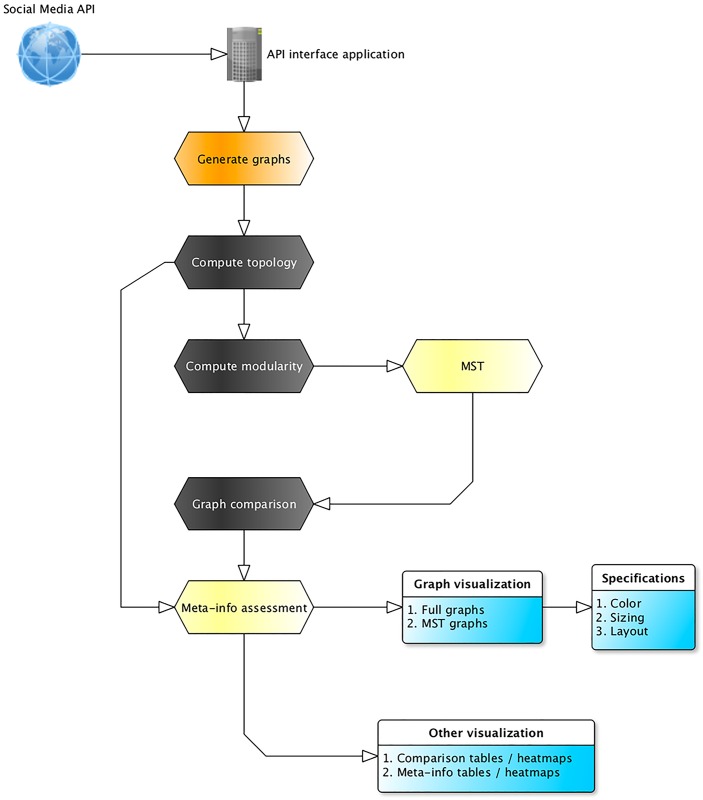
Method Overview.

An overview of the method is presented in Fig 1.

## Results

In the following sections, we describe the properties and structure (see Table 1) of each network graph. Taken collectively, the stakeholder networks demonstrate differences based on the types of stakeholder communities within the network (e.g. branches of the seeded network, awareness partners, and bloggers). Furthermore, each of these networks exhibit a mental-health related stakeholder community (e.g. suicide prevention, support for carers and youth-centric mental health services).

**Table 1:** Network Structure for each Organization.

Page	Nodes	Edges	Average Degree	Modularity (@ default resolution 1.0)
Mental Health America	216	2372	10.981	0.236
Mind	101	630	6.238	0.26
Beyond Blue	262	2598	9.916	0.257

### Network A: Mental Health America (USA)

**Fig 2:**
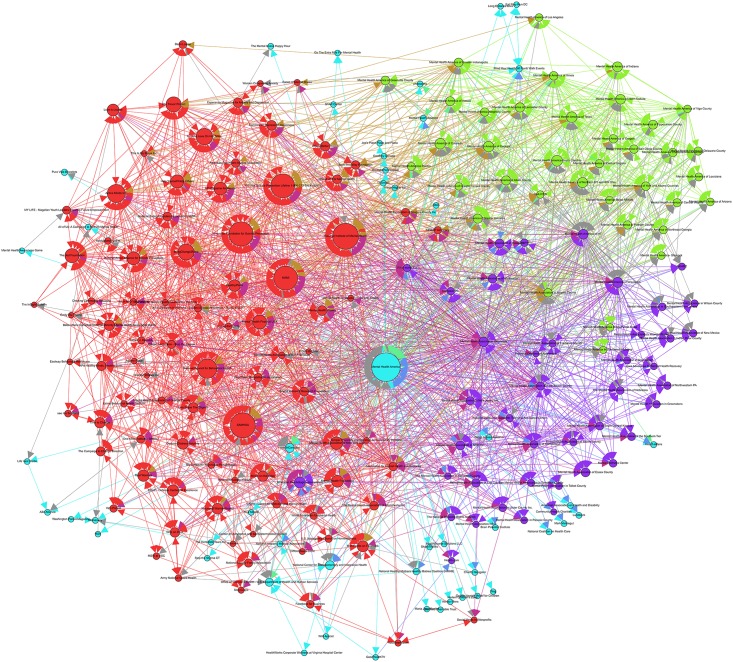
Network A visualization.

Network A, seeded from the node Mental Health America, comprised 216 nodes connected by 2372 edges with an average degree of 10.981 (see Table 1). This network graph partitions into four modules with a modularity score of 0.236: one core community (in red), two similar sized major communities (purple and green), and a more disparate community of nodes (light blue). As shown in Fig 2, a large section of the network is homogenous in nature, such that two organizations—Mental Health America and Mental Health Association—are sectioned by the geographical (state and county) location of the organization (i.e. Mental Health America of Wisconsin), and also dominate the core community.

Aside from these organization-based connections, the results indicate that a majority of the higher ranked nodes were located in one community (as seen in purple), which is composed of both not-for-profit and government led mental health organizations. We found that suicide-specific organizations (i.e. Active Minds, American Foundation for Suicide Intervention and National Suicide Prevention Lifeline) and broad-spectrum mental health organizations (i.e. SAMHSA, NAMI, and National Institute of Mental Health) were the most important within the local network. Further, on the surface, it was challenging to discern the formal and informal relationships (i.e. sponsorship and fundraising) between the nodes (i.e. organizations) within this network (Fig 2). This is in contrast with the networks of Mind and Beyond Blue (as seen below), which clearly demonstrate their integration with nodes in their network via branding and promotion.

### Network B: Mind (UK)

**Fig 3:**
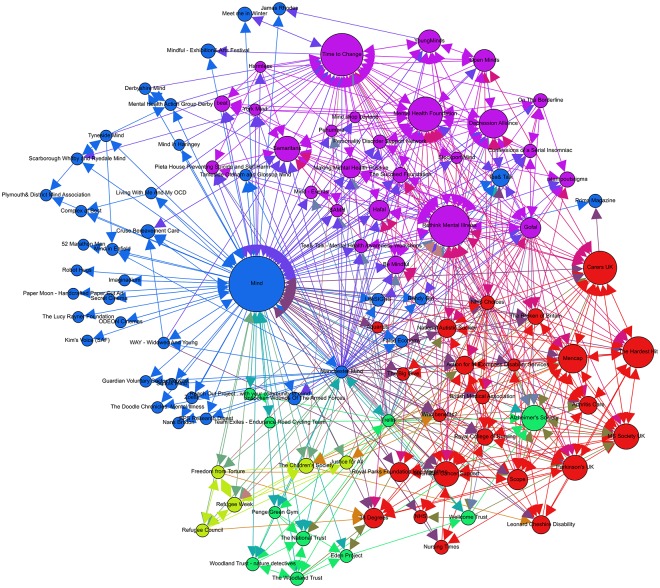
Network B visualization.

Network B, seeded from the node Mind, comprised 101 nodes connected by 630 edges with an average degree of 6.238 (see Table 1). This network graph partitions into five modules with a modularity score of 0.260. We found that Mind stands out as the mental health not-for-profit organization with the greatest number of associations with bloggers and community campaigners (see Fig 3) across its global network. These include include Confessions of a Serial Insomniac, The Broken of Britain and 38 Degrees. In Fig 3, we also observe that the three largest communities are comparable in size (purple, red and blue). The core community can be viewed close to the center of the global network; however, it does not include any nodes that have high ranks. More disparate nodes feature towards the bottom of the visualization (lime and light green communities).

Nodes with higher ranks in the network are visualized in the second two largest communities. These two communities are composed of mental health organizations (as seen in purple), and diverse health-related organizations (as seen in red) such as Multiple Sclerosis and Alzheimer’s Society. Among the mental health organizations, Time to Change, Rethink Mental Illness and Mental Health Foundation were the most important within the local network. Interestingly, Time to Change, a campaign body for mental health stigma and discrimination, is an organization that is not only led by Mind and Rethink Mental Illness, but also has strong ties through areas of awareness building and campaign development to smaller nodes in the periphery of the network. Among the diverse health-related organizations, Carers UK, Mencap and The Hardest Hit emerge as the most central in the network. With the focus of these organizations concentrated on the rights and improvements to well-being for individuals suffering from disability and their carers.

### Network C: Beyond Blue (Australia)

**Fig 4:**
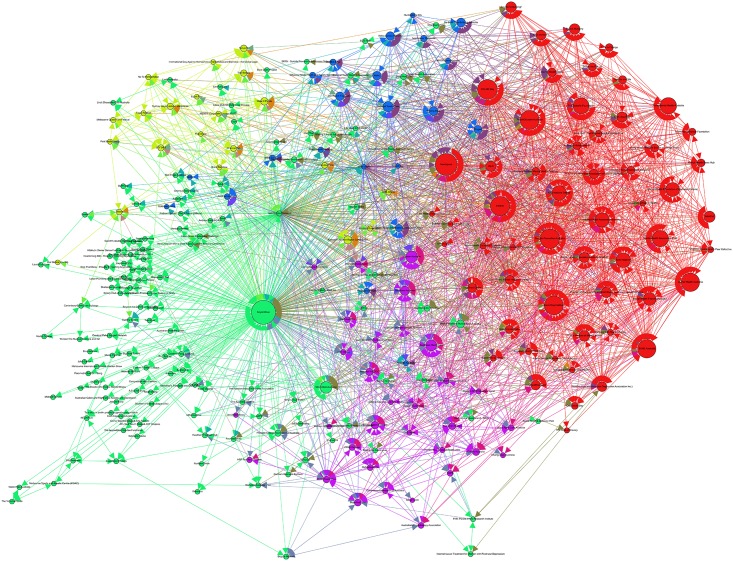
Network C visualization.

Network C, seeded from the node Beyond Blue, comprised 262 nodes connected by 2598 edges with an average degree of 9.916 (see Table 1). This network graph also partitions into five modules with a modularity score of 0.257: one core community (red), a second major community (green) and three smaller communities (purple, lime and blue). The results indicate that Beyond Blue has the most prominent associations with its collaborating awareness partners (see Fig 4) within its local network, particularly with Movember Australia (Men’s Health) and Mental Health in Multicultural Australia. These collaborating partners varied from celebrity endorsers to community and caused based not-for-profit organizations; with each of these partners working towards building awareness, advocating and raising funds for the programs and initiatives directed by Beyond Blue.

Outside the main community, we can see that a large portion of mental health organizations (or nodes) with the highest rank are contextually from a program and policy based mental health community. Among this policy and program based mental health organizations, the most collaborative nodes in this community can be divided into youth (i.e. Headspace, Reach Out and Black Dog Institute), mental health policy (i.e. Sane, Mental Health Australia and Rural Mental Health Australia), and suicide awareness and counseling (i.e. R U OK, Suicide Prevention and Lifeline) nodes. Accordingly, these nodes are deemed influential both in their local and global network—being strategic bodies within their sector of mental health—as well as in connecting varying issues and initiatives within the mental health eco-system.

### Minimum Spanning Trees (MST)

**Fig 5:**
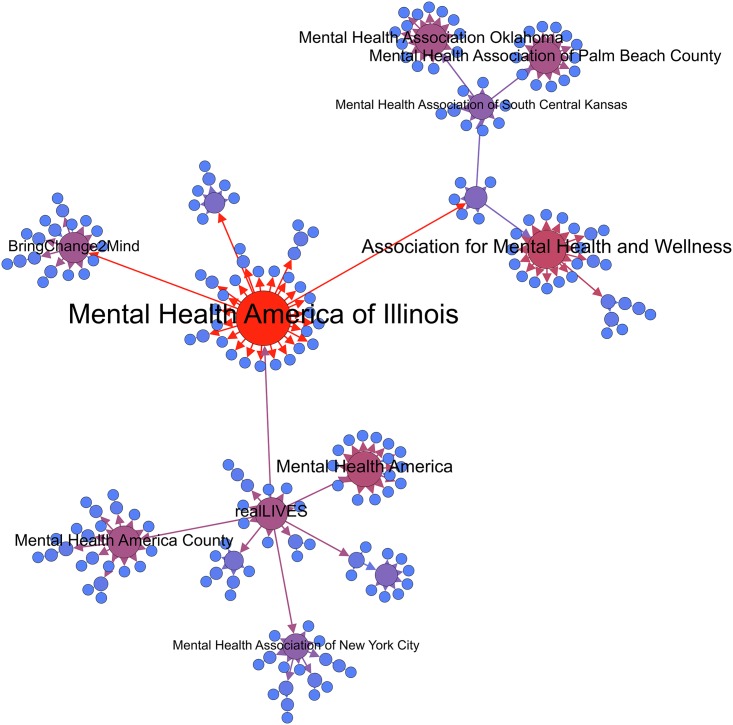
MST MHA visualizations.

The implementation of the MST procedure enabled the analysis of the fundamental structure across the three networks, highlighting the shortest path solution between the nodes, as well as the identification of hubs within each of the networks. The MHA network (see Fig 5) contained one central hub (in red) and seven major hubs (in dark purple), with the hubs in the network sectioned by geographical location of the organization. Mental Health America Illinois (MHAI) was identified as the central hub of the network, which can be contributed to the long standing operation of the organization in its geographical location. Specifically, this institution was one of the first developed by the community-based not-for-profit in 1909, with MHAI regarded as leading the way for awareness and reform in mental health care in Illinois [52].

**Fig 6:**
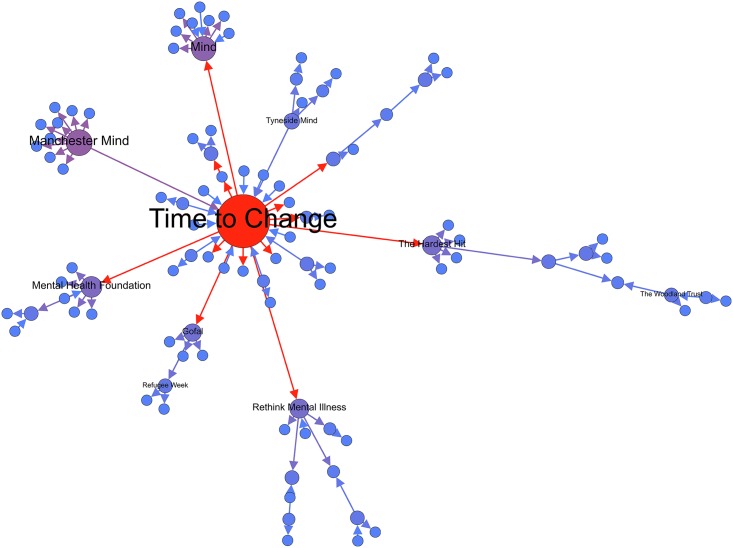
Mind MST visualization.

**Fig 7:**
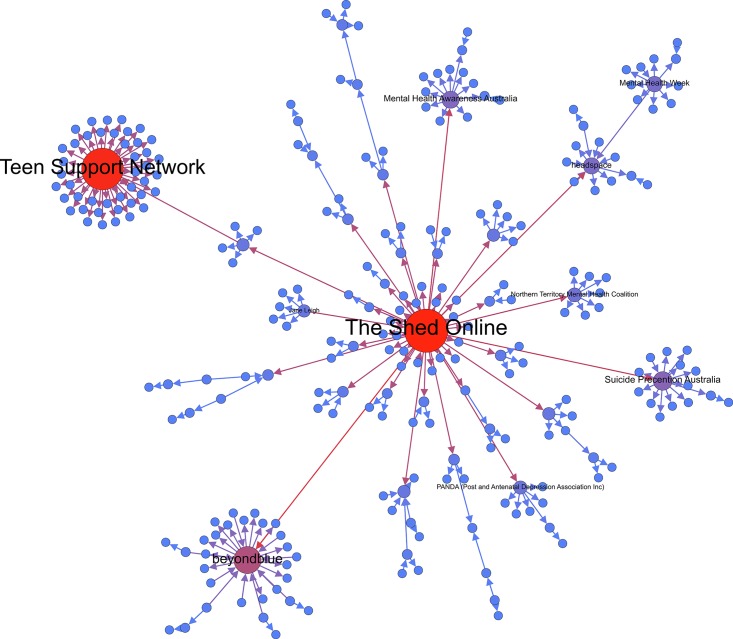
Beyond Blue MST visualization.

The MST visualization in Fig 6 demonstrated that the Mind network had one central hub (in red) and two minor hubs (in light purple) the network. Finally, among the Beyond Blue network (see Fig 7), the results indicate that there are two central hubs (in red) hubs and one major hub (in dark purple) connecting the nodes within the network. The central hubs within this network target two varying market segments for mental health including youth (Teen Support Network) and men’s mental health (The Shed Online, which is an initiative developed by Beyond Blue). Interestingly, across the three MST network visualizations, we can observe that the seeded mental health organization is not the central hub within the network. Rather, it is the not-for-profit initiatives and organizations developed by the seeded organization that are most central. For example, in the MST visualization for Mind, Time to Change—an anti-stigma mental health initiative developed and led by Mind and Rethink Mental illness—is the central hub in the network.

### Pairwise Network Comparison

As discussed, the output from the pairwise network comparison implemented with GraphCrunch 2 allows us to report, among other measures, GDD agreement (arithmetic mean), and RGF distance, to assess local-level topological similarity—along with Pearson and Spearman correlations of network degree distribution and path difference statistics, to supplement this information. The results of this analysis are presented in Table 2.

**Table 2:** Pairwise Network Comparison Results.

Network 1	Network 2	GDD amean	RGF dist	Degdist Pearson	Degdist Spearman	Path diff	Path diff %
MHA_full	MHA_MST	0.67	16.14	0.35	0.45	3.09	1.62
MHA_full	Mind_Full	0.69	1.81	0.79	0.87	0.01	0.01
MHA_full	Mind_MST	0.67	16.20	0.35	0.40	2.13	1.12
MHA_full	Beyond_Blue_Full	0.65	0.80	0.75	0.79	0.03	0.01
MHA_full	Beyond_Blue_MST	0.67	16.12	0.32	0.45	2.26	1.18
MHA_MST	Mind_Full	0.65	14.60	0.05	0.54	3.10	0.62
MHA_MST	Mind_MST	0.95	0.17	0.99	0.70	0.96	0.19
MHA_MST	Beyond_Blue_Full	0.62	15.68	0.20	0.44	3.07	0.61
MHA_MST	Beyond_Blue_MST	0.95	0.17	1.00	0.58	0.83	0.17
Mind_Full	Mind_MST	0.65	14.56	-0.08	0.22	2.15	1.13
Mind_Full	Beyond_Blue_Full	0.67	1.20	0.78	0.82	0.04	0.02
Mind_Full	Beyond_Blue_MST	0.65	14.46	0.04	0.31	2.28	1.20
Mind_MST	Beyond_Blue_Full	0.62	15.70	0.18	0.66	2.11	0.52
Mind_MST	Beyond_Blue_MST	0.94	0.14	0.99	0.74	0.13	0.03
Beyond_Blue_Full	Beyond_Blue_MST	0.62	15.60	0.19	0.83	2.24	1.15

Evidence for structural similarity between a pair of networks at a local level exists where GDD (0–1) is closer to 1 and RGF is closer to 0 [47]. Thus, these two values ‘mirror’ each other where local-level structural similarity exists. Table 2 also shows us how structurally similar or dissimilar the full networks are, relative to their reduced MST forms (e.g. Beyond Blue full and MST graphs score only 0.62 for GDD).

A visual inspection shows that the pairs of MST graphs are most similar in the full set of six networks. This finding is to be expected, given the comparative structural simplicity of the MST graphs as compared with the full networks. The GDD scores provide a similarity ‘rank’, with values ranging across networks from 0.62 to 0.95 and RGF scores from 0.14 to 16.2, which indicates substantial variation between networks in terms of local topology. However, overall, each of the pairs of full networks exhibit very similar characteristics at both the local and global levels.

The results show that MHA and Mind network pairing have the best GDD score (GDD = 0.69), but a lower performing RGF score (RGF = 1.81), and the highest degree distribution correlation scores (Pearson = 0.79, Spearman = 0.87). Conversely, the MHA and Beyond Blue network pairing has a lower GDD score (GDD = 0.65), yet has the best RGF score of the three full network pairs (RGF = 0.80). This latter network pairing also has the lowest degree distribution correlation scores (Pearson = 0.75, Spearman = 0.79). Alternatively, the Mind and Beyond Blue full network pairing have the second best GDD score (GDD = 0.67), a mid-range RGF score (RGF = 0.80), and a mid-range degree distribution correlation scores (Pearson = 0.78, Spearman = 0.82). As additional supplementary topological information, the path difference statistics correspond closely with the degree distribution scores for all network pairings. This quick check of topological structure provides an important basis for more detailed analysis of the networks. In practice, this would allow marketing managers to understand the fundamental comparability of their brand’s network versus those of other organizations, before pursuing more focused analysis.

### Graph Meta-information

Having examined the structural properties of each network, we now turn attention to embedded node meta-information (i.e. stakeholders) for each network in the form of linked social media metrics. This is presented in Table 3. We focus on page ‘likes’ as a proxy for the size of a page’s external audience and influence within a broader social media stakeholder network. We also supplement this analysis by examining website’s of the seed nodes, and of, nodes identified with high ‘like’ counts to identify and verify past and present relationships.

**Table 3:** Node Meta-information Assessment.

Mental Health America						Mind						Beyond Blue					
ID	Likes	indeg	outdeg	deg	EVC	ID	Likes	indeg	outdeg	deg	EVC	ID	Likes	indeg	outdeg	deg	EVC
Demi Lovat…	36207485	8	0	8	0.15	Zoella	2108334	2	0	2	0.10	Norton	1279676	1	2	3	0.05
Facebook f…	9366222	9	1	10	0.14	Macmillan…	553807	15	1	16	0.37	Foxtel	855854	4	2	6	0.06
Health.com	3106893	5	0	5	0.08	ODEON Cine…	472942	1	1	2	0.09	triple j	834439	18	0	18	0.24
Wounded Wa…	2832079	20	0	20	0.27	The Nation…	287986	7	1	8	0.16	Chet Faker	657819	1	0	1	0.05
To Write L…	1395696	35	3	38	0.50	Secret Cin…	252126	1	1	2	0.09	The Random…	642020	3	0	3	0.06
Non-Profit…	993451	26	1	27	0.28	Time to Ch…	191797	37	13	50	0.73	beyondblue…	373463	133	261	394	1.00
philosophy	572738	2	0	2	0.07	Mind	175839	55	100	155	1.00	Daniel Mor…	318062	8	0	8	0.10
HealthCare…	469318	13	2	15	0.19	Alzheimer…	169092	13	2	15	0.38	R U OK Day	278912	72	16	88	0.79
It Gets Be…	379183	17	2	19	0.22	38 Degrees	156212	11	0	11	0.27	Hawthorn F…	258903	6	2	8	0.06
Born This…	324871	11	0	11	0.17	Pieta Hous…	155097	5	3	8	0.13	Julia Gill…	248751	11	4	15	0.08
Momastery	324203	5	0	5	0.07	Rethink Me…	146636	33	14	47	0.70	Canterbury…	247311	4	4	8	0.06
Pura Vida…	321607	2	0	2	0.08	The Woodla…	127948	5	6	11	0.14	The Anxiet…	227100	22	46	68	0.27
The Trevor…	304034	33	6	39	0.45	Mental Hea…	106278	26	8	34	0.57	Lindt Aust…	226624	3	2	5	0.05
American P…	292985	26	3	29	0.28	Eden Proje…	85465	4	1	5	0.12	Channel Te…	219837	11	1	12	0.11
National S…	206228	55	18	73	0.81	NHS Choice…	74380	4	31	35	0.15	Optus	212652	5	3	8	0.06
Time to Ch…	191797	20	3	23	0.26	Royal Coll…	47936	8	10	18	0.20	Sydney Swa…	211458	7	0	7	0.07
Brain & Be…	179422	16	54	70	0.19	Nursing Ti…	43702	4	2	6	0.15	NEON Run	198064	4	3	7	0.05
National I…	172502	72	21	93	0.89	Scope	40369	10	15	25	0.27	Laura Dund…	195039	2	0	2	0.05
NAMI	168556	71	1	72	0.81	Samaritans…	37389	16	4	20	0.38	Time to Ch…	191797	12	0	12	0.20
Love is Lo…	156013	16	7	23	0.31	MS Society…	37342	14	8	22	0.38	Telstra	176189	10	6	16	0.09
American F…	148994	57	4	61	0.82	Woodland T…	37156	5	2	7	0.13	Sydney FC	131644	3	0	3	0.07
HealthyPla…	123018	23	7	30	0.30	NHS	31821	4	0	4	0.13	Student Ed…	127749	7	8	15	0.12
StoryCorps	119062	4	0	4	0.08	Parkinson…	31224	13	10	23	0.37	Australian…	118591	35	2	37	0.36
Mental Hea…	106278	27	1	28	0.32	Carers UK	28368	17	12	29	0.54	Waratahs	117527	2	1	3	0.05
Mental Hea…	95636	102	215	317	1.00	Mencap	25975	16	5	21	0.44	Bupa Austr…	108782	13	7	20	0.15

S1 Fig shows that for Mental Health America, the node Facebook pages of Wounded Warrior Project (charity), Facebook for business, and Demi Lovato (Musician) have the highest page ‘like’ counts within the MHA network graph. However, according to their degree and Eigenvector centrality rankings, they are comparatively less embedded than many other brand pages with smaller external audiences. The existence of reciprocal page ‘likes’ can be assessed according to the in-degree and out-degree counts, where an out-degree score of zero against a positive in-degree score indicates that a brand page does not reciprocate a page ‘like’ (i.e. does not ‘like a page back’).

S2 Fig also shows that for Mind, the results indicate that node Facebook pages of Zoella (fashion and lifestyle blogger), Macmillian Cancer Support, and ODEON Cinema had the highest ‘like’ counts. As with the MHA network graph, we can see that these scores do not correspond with the highest ranks for degree and Eigenvector centrality. This suggests that there is scope for Mind to leverage and/or re-engage with these connections with these stakeholders (nodes) which possess large and influential external audiences. For instance, an examination of the relationship between Mind and Zoella (see S2 Fig) via a website analysis identifies an existing partnership, where Zoella acts as a digital ambassador for Mind in launching the initiative #DontPanicButton to raise awareness of anxiety and panic attacks in young adults [53]. Although Zoella has over 8 million subscribers to her Vlog website and over 2.5 million followers on Facebook [53, 54], Mind has yet to leverage this external audience by posting content on the Mind Facebook page *and* Zoella’s Facebook page in order to access larger networks of potential followers.

For Beyond Blue, we observe that Norton (anti-virus and security software), Foxtel (cable TV), Triple J (youth radio station), and Chet Faker (musician) had the highest ‘like’ counts in this network. As with the previous network graphs, the ratio of out-degree to in-degree counts for this network reveals a number of page ‘like’ relationships that have not been reciprocated via Facebook platform. For instance, in analyzing the relationship between Norton and Beyond Blue via their website (see S3 Fig), a relationship was established between these stakeholders and high profile athlete Jarrod Hayne to promote awareness of online security and cyber bulling to teenagers and young adults [55]. This public service announcement takes place with content available on the website platform, however, has not been reflected in the Facebook platform. Therefore, opportunities exist to leverage the networks of Norton and Jarrod Hayne via social media to expand reach, and enable cross promotion and brand awareness opportunities for Beyond Blue which would also provide mutual benefit for Norton and Jarrod Hayne.

This relatively simple phase of analysis shows that, across all three networks, there exist valuable nodes that have not been more tightly integrated via (a) reciprocation of social media relationships, and (b) the building of more connections with other nodes that also share connections with the seed organization. We can assume that these pages with high ‘like’ counts have large external audiences, high user engagement levels and also exert a substantial influence within a larger network of potentially relevant individuals. By simply comparing relevant social media metrics against network topology measures, opportunities for leveraging such nodes can be highlighted.

## Discussion

We have illustrated how actively scanning stakeholder networks presents opportunities for not-for-profit mental health organizations to enhance relationship management effectiveness. For resource-constrained organizations (i.e. not-for-profit organizations), social media affords (not-for-profit) marketers the ability to identify and manage valuable stakeholder relationships in a less resource-intensive manner [9]. To this end, our findings provide novel insights for marketing and social media managers to: (1) implement a useful, feasible and sustainable approach to small-scale stakeholder network analysis; (2) identify and target relevant (and non-relevant) stakeholders for the development of formal and informal reciprocal relationships by leveraging resources to enhance marketing effectiveness (i.e. accessing broader networks to increase reach and exposure); (3) make informed resource allocation decisions to optimize and focus marketing activities towards the most relevant stakeholders to build formal and/or informal reciprocal relationships. Further, the proposed approach is flexible and can be applied to similar or different contexts (e.g. other health services, charities, NGOs, education, media, telecommunications, professional services and commercial brands).

Our initial assessment of network graph properties and structure revealed interesting differences between the network graphs in the three international markets selected. We have been able to provide a snapshot of past and present partnerships with stakeholders. As well as identifying missed opportunities for future collaboration with relevant stakeholders. Finally, we have demonstrated how network graph reduction techniques can be used to help in this process. For instance, Mind has a formal reciprocal relationship with Rethink Mental Illness; however, it does not have a reciprocal relationship with Zoella (a fashion and lifestyle blogger). Whereby Zoella was found to be a key stakeholder within their network. As such, Mind has missed an opportunity to collaborate, and leverage from, Zoella’s network of followers.

By examining network graph meta-information, we have also been able to demonstrate that not-for-profit marketers should consider the ‘role’ stakeholder’s play in networks relative to their importance in their respective external networks. Each of the networks revealed stakeholders with large external audiences that were not necessarily tightly integrated in the immediate network. Therefore, it appears that analysis of social media stakeholder networks—even at a small-scale—can provide a plethora of potentially useful insights.

In terms of feasibility, adopting our approach would be a viable inclusion in the social media activities of not-for-profit health organizations owing to the relative simplicity, but demonstrable value of the techniques presented. The data extraction procedure and analysis techniques presented here are all implemented using existing free-to-use GUI software packages, making the approaches both cost-effective and accessible.

We believe that studying stakeholder networks using social media data is an important avenue for future research, especially in the context of profitable service organizations and not-for-profits, where organizations manage rich networks with a variety of stakeholders. To foster further research around this fruitful line of enquiry, we propose four possible avenues, as follows.

First, it would be interesting to extend the network scale to include an extra degree of connections, and repeat this analysis. This suggestion would offer more insight into ‘chains’ of stakeholder relationships within extended networks, including the potential capture of longitudinal network dynamics, as well as the investigation of endogenous and exogenous mechanisms of network evolution [56, 57]. Such research endeavors would assist in uncovering the contextually determined factors that shape stakeholder investment and outcomes in service networks [14].

A second possible research avenue could involve combining data from multiple social media sources, or other data sources containing information pertaining to stakeholder networks. This type of extension could be used to help develop weighted composite metrics of social media stakeholder engagement across platforms. Such an extension could focus specifically on the meta-information phase of our study, where several social media metrics could be compared against numerous network topology measures. Researchers could also study the specific meaning and ‘value’ of different social media metrics in this context. Along these lines, future research should incorporate other widely used measures of topological assessment such as degree-based entropy [58, 59] and alternative approaches to network comparison (see: [50] for further reading).

Third, we recommend that researchers combine brand network data with topic network data [60], to study the content stakeholders discuss and share on social media within networks. This line of enquiry could also build upon recent research to included patient perceptions [61], which would be invaluable in extending the stakeholder network perspective to include more customer insights, ultimately highlighting the role of stakeholder networks and their structure in value co-creation.

Finally, the development of network analytics software tools tailored to the needs of marketing where stakeholder networks are prevalent—as is the case for not-for-profit marketers—represents an important priority for future research. Such research would investigate ways to collect, process and present this data on an ongoing basis [62], to generate insights that help marketers craft and optimize marketing strategies with their key stakeholders in their network. As we have illustrated, such a process begins with selecting efficient and accessible tools to simplify intricate and detailed stakeholder networks in this context. Subsequent optimization efforts would then focus on ‘key’ stakeholder identification as a basis for (1) retrospective assessment of the success of past initiatives conducted between stakeholders, and (2) research and intelligence gathering around the current success and relevance of promotional activities being conducted by other stakeholders.

In sum, we believe our study will lead to the development of more research in the area of stakeholder networks in social media. We encourage future research to help profitable and not-for-profit services extract value from these networks.

## Supporting Information

S1 Fig MHA Graph Meta-Information Network. (TIF)

S2 Fig Mind Graph Meta-Information Network. (TIF)

S3 Fig Beyond Blue Graph Meta-Information Network. (TIF)
