# Supplementary figures and images for: An Examination of Not-For-Profit Stakeholder Networks for Relationship Management: A Small-Scale Analysis on Social Media
Source: PLoS One. 2016 Oct 6;11(10):e0163914. doi: 10.1371/journal.pone.0163914 (PMC5053609; doi:10.1371/journal.pone.0163914)

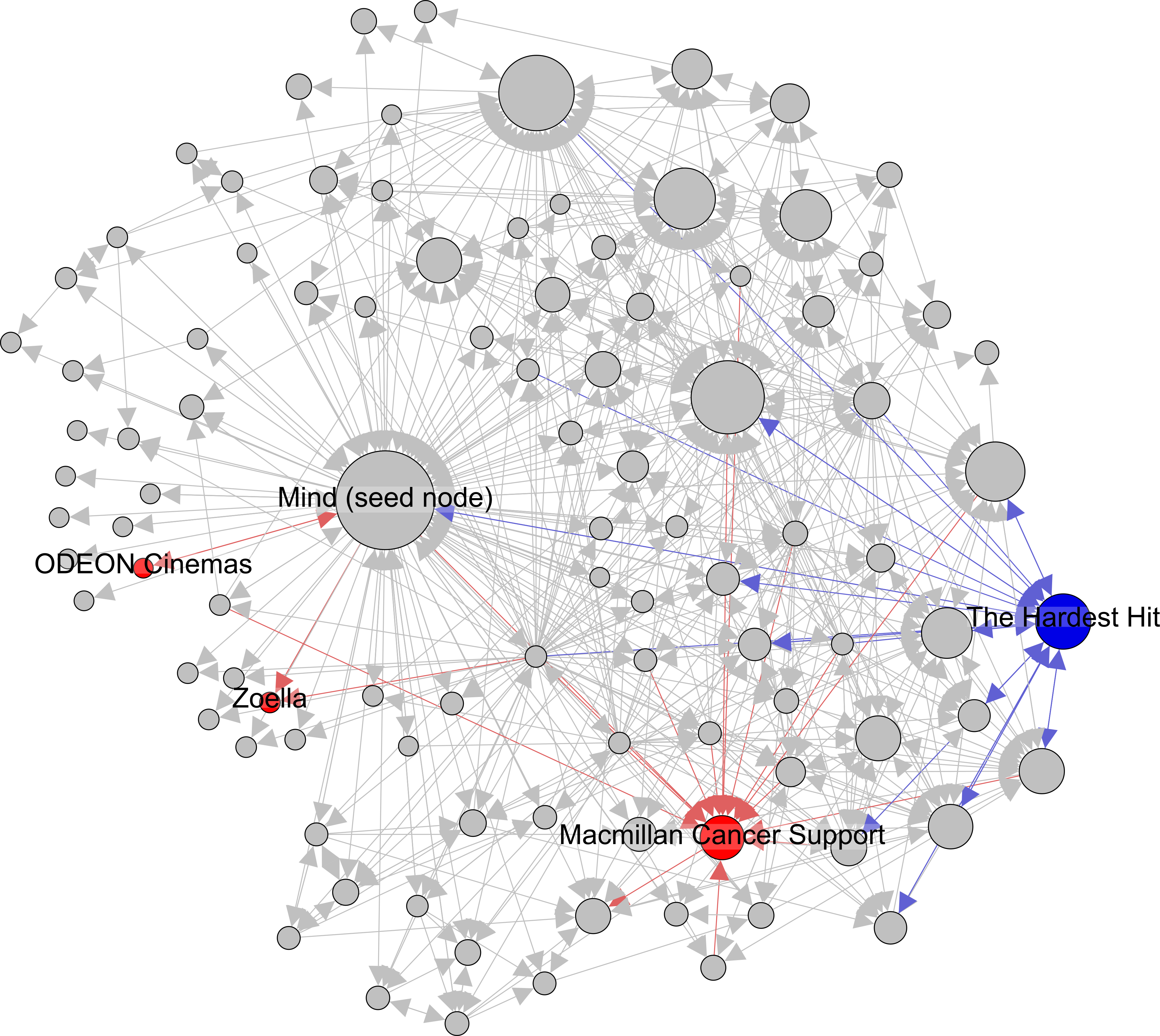

Supplement: S2 Fig — (TIF) [file pone.0163914.s002.tif]
